# Supplementary material for: Chemical constituents, antibacterial, acaricidal and anti-inflammatory activities of the essential oils from four Rhododendron species
Source: Front Vet Sci. 2022 Aug 10;9:882060. doi: 10.3389/fvets.2022.882060 (PMC9399923; doi:10.3389/fvets.2022.882060)
Supplement: Supplementary Table S1 — The identified compounds of the essential oils from four Rhododendron species. [file Data_Sheet_1.zip › 22-7-24/Manuscript 6-9revision.docx]

Chemical constituents, antibacterial, acaricidal and anti-inflammatory activities of the essential oils from four *Rhododendron* species

Jian He^a,b#^, Xiaofei Shang^a,b#^, Lixia Dai^a^, Xiaorong Yang^a^, Bing Li^a^, Yanming Wei^b*^, Jiyu Zhang^a,b*^, Hu Pan^a^.

^a^ Key Laboratory of New Animal Drug Project, Gansu Province, Key Laboratory of Veterinary Pharmaceutical Development of Ministry of Agriculture, Lanzhou Institute of Husbandry and Pharmaceutical Sciences, Chinese Academy of Agricultural Sciences, Lanzhou 730050, P.R. China.

^b^ College of Veterinary Medicines, Gansu Agricultural University, Lanzhou 730070, P.R. China

*****To whom corresponding author should be addressed.

E-mail: shangxiaofei@caas.cn.

**Abstract**

As the ornamental plants and traditional medicines, *Rhododendron przewalskii*, *R. anthopogonoides*, *R. thymifolium*, and *R. capitatum* are widely distributed in western China. In this paper, the essential oils from these four species were extracted by supercritical extraction and the components were analyzed using headspace solid phase microextraction combined with gas chromatography-mass spectrometry (HS-SPME-GC-MS), the antibacterial, acaricidal and anti-inflammatory activities were investigated. Results showed that *R. thymifolium* (RTEO) contained the highest yield of 0.99% with 246 compounds, followed by *R. capitatum* (RCEO, 0.81%) with 290 chemicals, *R. anthopogonoides* (RAEO, 0.57%) with 302 compounds and *R. przewalskii* (RPEO, 0.30%) with 294 components. They also exhibited the safety at given doses and have the anti-inflammatory *in vitro* and *in vivo* tests via inhibiting the cytokines productions, the acaricidal and antibacterial activities also were found. 4-Hydroxy-3-methylacetophenone from RPEO, α-pinene and β-pinene from RAEO, β-farnesene and germacrone from RTEO, and benzylacetone from RCEO, as main and active components, inhibited the NO content in RAW 264.7 cells induced by LPS. These results indicated that four essential oils have certain medicinal value and laid the foundation for the development of these species as raw materials for the pharmaceutical and perfume industries.

**Keywords:**

Acaricidal activity, Anti-inflammatory activity; α-Pinene; Essential oils, *Rhododendron* species

1. **Introduction**

As an important component and secondary metabolite of medicinal plants, essential oils from the stems, leaves, flowers, roots and fruits of aromatic plants play very important roles both economically and scientifically worldwide. Essential oils of various plants are rich in bioactive substances, including monoterpenes, sesquiterpenes, and their derivatives, such as aldehydes and phenols, simple phenylpropanoids and others, and these components vary for different species and in different seasons. Reports have shown that they possess biological properties, such as anti-inflammatory, antioxidant, antimicrobial, antifungal, acaricidal and insecticidal activities (1,2,3,4). Due to their low toxicity to humans, capacity for further degradation, and low environmental impact, plant essential oils have been widely applied in the pharmaceutical, perfume, an cosmetics industries and in plant protection and veterinary medicine.

*Rhododendron* (Ericaceae family) is one of the largest genera of vascular plants, with approximately 967 species, and is widely distributed in the Northern Hemisphere. Some species have been used in China, India, Europe and North America as traditional medicines for treating inflammation, pain, skin and alimentary canal diseases ,some species are cultivated as ornamental plants for horticulture and economic crops for the pharmaceutical industry (5). Modern studies have shown that this genus consists of flavonoids, diterpenoids, iridoid glycosides and sesquiterpenoids (6). Among these, essential oils were thought to be the active components of *Rhododendron* species*.* Essential oils from *R. tomentosum* are thought to be a source of potential antiarthritic drugs for antiproliferative and proapoptotic activities toward CD4 and CD8 T cells, synovial-infiltrating monocytes/macrophages and fibroblast-like synovial cells (7).There are more than 700 *Rhododendron* species in China, most of which are widely distributed in the Himalayas (8). As the dominant species in this region, *Rhododendron przewalskii* Maxim., *R. anthopogonoides* Maxim., *R. thymifolium* Maxim., and *R. capitatum* Maxim. are distributed and planted in western China, especially in Gansu, Qinghai and Sichuan provinces (9,10). They are widely used as traditional Tibetan medicines to treat chronic bronchitis and cough, as well as nectariferous medicines (11). From *R. thymifolium,* 14 chemical components were identified from the essential oil, such as germacrone (20.83%), γ-elemene (11.10%), and selina-3,7(11)-diene (6.18%), and their insecticidal activities against *Liposcelis bostrychophila* or *Tribolium castaneum* have been proven (9). The essential oils from *R. anthopogonoides*, *R. thymifolium*, and *R. capitatum* have good efficacy in relieving cough, function as expectorants, and inhibit *Staphylococcus aureus* (12). However, the chemical constituents and the antimicrobial and anti-inflammatory activities of the essential oils from four *Rhododendron* species have not been studied and compared comprehensively. In this paper, we aimed to identify the components of four kinds of *Rhododendron* essential oils, clarify and compare their biological activities, and to determine whether the main components are related to biological activity. This study will lay the foundation for the future development of the essential oils from four Rhododendron species in pharmacy.

1. **Materials and methods**

*2.1. Plant material*

The fresh leaves of *R. przewalskii* (5 kg), *R. anthopogonoides* (5 kg), *R. thymifolium* (5 kg) and *R. capitatum* (5 kg) were collected from the northern slope of a mountain near the Zhuaxixiulong region of Tianzhu County, Gansu Province, China (37°11.4′ N latitude, 102°46.1′ E longitude, 2922 m), in June 2020. The species were identified by Prof. Chaoying Luo from Lanzhou Institute of Husbandry and Pharmaceutical Sciences. All these voucher specimens were deposited at the Herbarium with the No. ZSY422-425. The collected leaves were shade-dried for the following tests.

*2.2.* *Essential oils extraction and HS-SPME-GC-MS analysis*

Essential oils of four species were separated by the Spe-ed SFE-2 supercritical carbon dioxide extraction technique (Applied Separations, USA) for 2 h at following conditions: CO_2_ pressure: 350Bar, cooling circulation system: 5℃, oven temperature: 40℃, valve temperature: 80℃, vessel temperature: 40℃, and the oils were obtained and kept in sealed glass vials (13). The sample (50 mg) with 10 μL of 2-octanol (10 mg/L stock in dH_2_O) as an internal standard was placed into a 20-mL bottle for analysis in a gas chromatograph system coupled with a spectrometer (GC-MS).

In the solid-phase microextraction (SPME) cycle of the PAL rail system, the incubation temperature was 60 °C, the preheating time was 15 min, the incubation time was 30 min, and the desorption time was 4 min. GC–MS analysis was performed using an Agilent 7890 gas chromatograph system coupled with a 5977B mass spectrometer. The system utilized a DB-Wax injected in Split Mode (50:1), helium was used as the carrier gas, the front inlet purge flow was 3 mL/min, and the gas flow rate through the column was 1 mL/min. The temperature of the injector was 250°C.The initial temperature was 40 °C for 4 min, raised to 245 °C at a rate of 5 °C/min, and maintained for 5 min. The injection, transfer line, ion source and quad temperatures were 250, 250, 230 and 150 °C, respectively. The energy was -70eV in electron impact mode. The mass spectrometry data were acquired in scan mode with an m/z range of 20-400 and a solvent delay of 0 min. Chroma TOF 4.3X software, produced by LECO Corporation, and the NIST database (version is 2.4,built in March 25 2020) were used for raw peak extraction, database filtering and calibration of the baseline, peak alignment, deconvolution analysis, peak identification, integration and spectrum matching of the peak area.

*2.3. Antimicrobial activity*

The antibacterial activities of the four essential oils were determined against three gram-negative bacteria, *Salmonella* (ATCC 14028), *Escherichia coli* (ATCC 25922), and *Pseudomonas aeruginosa* (ATCC 21625), and three gram-positive bacteria, *Staphylococcus aureus* (ATCC 25923) and *Listeria monocytogenes* (ATCC 21529), which were purchased from ATCC (U.S.A.), and *Enterococcus faecalis* (BNCC 102668), which was purchased from BeNa Culture Collection (BNCC, China). These bacterial strains were grown for 24 h in Mueller-Hinton broth (MHB) at 37 °C. The minimum inhibitory (MIC) values of the four essential oils against the above human pathogenic bacteria were investigated. Ampicillin sodium and Streptomycin sulfate were used as references (Solarbio Science & Technology Co., China). All tests were performed three times (14).

*2.4. Acaricidal activity*

*Psoroptes cuniculi* were collected from the external auditory canals of rabbits naturally infected with mites, which were provided by a rabbit farm in Yuzhong of Lanzhou City, China. The rabbits were treated immediately after collecting all mites. Acaricidal activity was determined according to a previously described method (15). Specifically, 200 μL of four essential oils diluted in 10% DMSO were added to plates, and the excess liquid was absorbed. 10% DMSO was applied to the untreated group. Then, 20 adult mites were placed in each plate, all of which were kept at 25 °C and 75% relative humidity for 24 h. The number of dead mites was observed under a microscope. Each sample was repeated three times.

*2.5. Anti-inflammatory activity*

*2.5.1. In vivo anti-inflammatory activity*

*2.5.1.1. Mice*

BALB/c mice (18-22 g) were obtained from the experimental animal center of Lanzhou Veterinary Research Institute, CAAS. Diets and water were provided ad libitum, with light for 12 h per 24 h. All animal tests were performed according to the guidelines of the Ethics Committee of Medical Sciences.

*2.5.1.2. Carrageenan-induced paw edema in mice*

Mice were randomly divided into 6 groups: a model group (normal saline), four essential oil groups (200 mg/kg) and a positive control group administered dexamethasone (DEX, 2 mg/kg). Each group contained 6 mice. Before the experiment, each group was given the corresponding essential oils by gavage, and 1 h later, 25 μL of 2% carrageenan was injected into the right hind paw. The thickness levels of the left and right soles of mice were accurately measured with Vernier calipers after 5 h, and the percentage of inhibition was calculated (16).

*2.5.1.3. Xylene-induced ear edema*

Before the test, four essential oils were given to each group by gavage. After 1 h, the left ears of the mice were evenly coated with 30 μL of xylene. Two hours later, mice were sacrificed, both ears were cut off along the root, and patches were obtained at the same parts of the left and right auricles with a 7-mm-diameter punch. All patches were weighed accurately, and the edema inhibitory rate was calculated (16).

*2.5.2. In vitro anti-inflammatory activity*

The primary mouse macrophage RAW 264.7 cell line was obtained from Prof. Zhang’s lab., Lanzhou Institute of Husbandry and Pharmaceutical Sciences, CAAS (Lanzhou, China). RAW264.7 cells (500 μL) in the logarithmic growth stage were inoculated on 24-well plates at a concentration of 1×10^5^/mL. The medium was removed after culture at 37 °C for 24 hours, and then equal amounts of medium containing four essential oils (compounds) at different concentrations were added. One hour later, 5 μL of LPS (0.5 mg/mL) was added to each well except the blank control group, and the cells were cultured for another 24 h to induce inflammation. DEX (5 μg/mL) was used as a positive control. Finally, the cells were collected to measure the levels of nitric oxide (NO), interleukin-6 (IL-6) and superoxide dismutase (SOD) using enzyme-linked immunosorbent assay kits from Nanjingjiancheng Bio (NJJCBIO, China), and the absorbance was measured using a Multiskan Go Microplate Spectrophotometer (Thermo Scientific., U.S.A).

*2.6. Toxicity*

*2.6.1. Cytotoxicity*

The cytotoxicities of the four essential oils against RAW 264.7 cells were evaluated using the Cell Counting Kit-8 (CCK-8, ZETA Life, U.S.A.). RAW 264.7 cells (100 μL) at a density of 1×10^5^ cells/well were incubated in 96-well plates for 24 h, 10 μL of the four essential oils (250-15.63 μg/mL) or their main compounds were added and incubated for 24 h, respectively, and DMSO (0.1%) was used as a control. Then, CCK-8 agent (10 μL) was added for 30 min, and the absorbance was measured at 450 nm. Each sample was repeated three times, and cell viability was calculated.

*2.6.2. Acute toxicity*

The up-and-down method for acute toxicity testing was performed. The dose was increased from 2000 to 5000 mg/kg through the oral route of administration. The animals were observed continuously for behavioral changes for the first 4 h and then for mortality, if any presented, 24 h after the drug administration.

1. **Results**

*3.1. The essential oils of four* Rhododendron *species*

In this paper, the essential oils of four *Rhododendron* species were obtained using CO_2_-SFE. *R. thymifolium* (RTEO) contained the highest yield of 0.99% (w/w), followed by *R. capitatum* (RCEO, 0.81%), *R. anthopogonoides* (RAEO, 0.57%) and *R. przewalskii* (RPEO, 0.30%), as shown in Fig. 1a.

*3.2. Chemical composition of the essential oils*

In this paper, HS-SPME-GC-MS analysis was performed to enrich and then analyze the components of the essential oils in four *Rhododendron* species, and total ion flow diagrams were obtained, as shown in Fig. S1.

After screening the components based on spectral similarity values greater than 700, 302 chemicals were identified from RAEO, and the main compounds were α-pinene (8.47% of the oil), followed by humulene (5.94%), β-pinene (4.07%), o-cymene (3.45%) and myrtenol (3.11%). For RPEO, 294 components were identified, and the main components were phenylethyl alcohol (12.01%), 4-hydroxy-3-methylacetophenone (5.55%), benzyl alcohol (4.09%), calarene (2.96%), and 4-phenyl-2-butanol (2.66%). RTEO has appreciable contents of cyclohexanone, 5-ethenyl-5-methyl-4-(1-methylethenyl)-2-(1-methylethylidene)-(11.02%), β-farnesene (4.99%), γ-cadinene (4.83%), selina-3,7(11)-diene (3.93%), bisabolone (3.84%), as well as other 241 chemicals; finally, 290 compounds from RCEO were found, and the major constituents were benzylacetone (11.30%), benzene, 1-ethenyl-4-ethyl- (7.91%), γ-muurolene (7.12%), and α-selinene (4.50%) (Table S1). The top 30 compounds were list in Table 1.

55 common compounds were identified from these four essential oils, such as phenylethyl alcohol, acetic acid, hexanoic acid, 2-butanone, 4-phenyl-, linalool, and benzaldehyde (Fig. 1b, Table S2). However, although these species were collected from the same altitude and environment, more compounds were different, which was dependent on the different biosynthetic pathways and inheritances. Different chemicals may result in different activities and uses.

*3.3. Antibacterial activity*

This study showed that among the three gram-positive bacteria, they presented an inhibitory effect on *S. aureus*, and the MIC values were 0.26 mg/mL for RAEO and 1.0 mg/mL for RCEO and RTEO. The activity of RPEO was weak (MIC 4.1 mg/mL). However, four essential oils showed antibacterial activity against *P. aeruginosa* among only three gram-negative bacteria, especially RAEO, with its MIC value of 0.06 mg/mL (Table 2).

*3.4. Acaricidal activity*

To investigate the potential acaricidal activity of *Rhododendron* sp., the toxicity of the four essential oils against *Psoroptes cuniculi* was studied. As shown in Table 3, among the four essential oils, the acaricidal activity of the RAEO stands out, and the LC_50_ at 24 h was 1.59 mg/mL, followed by RPEO with an LC_50_ of 1.71 mg/mL. The LC_50_ of RTEO was 2.35 mg/mL, and RCEO had the poorest effect, with an LC_50_ of 4.10 mg/mL.

*3.5. Anti-inflammatory activity*

*3.5.1. Carrageenan-induced paw edema in mice*

As shown in Fig. 2c,d, compared with the model group, the essential oils (200 mg/kg) significantly reduced the paw edema of mice caused by carrageenan. Among them, RAEO and RPEO had the best effects, and their inhibition rates on foot edema were 60.47% and 58.59%, respectively, with no significant difference compared with the positive control drug dexamethasone, with an inhibition rate of 57.65%. The effects of RTEO and RCEO were obviously weaker than that of DEX (*P < 0.05*).

*3.5.2. Xylene-induced ear edema*

Subsequently, a xylene-induced ear edema test was used to evaluate the vascular permeability of essential oils, which was partially associated with substance P. The results showed that compared with the model group, the four essential oils notably reduced the mouse ear edema caused by xylene. The effect of RAEO was the best, with an inhibition of 68.55%, which was higher than that of 60.17% dexamethasone (Fig. 2e, f). There was no significant difference in the inhibitory effects among the groups.

*3.5.3. In vitro anti-inflammatory activity*

The results showed that the level of NO secreted in the model group was significantly higher than that in the control group (*P<0.01*); however, after essential oil treatment, the NO secretion level decreased markedly in a concentration- dependent manner in the model group (*P<0.01*). The activities of the essential oils were as follows: RCEO> RTEO> RAEO>RPEO at the concentration of 12.5 μg/mL (Fig. 2g). Essential oils can significantly reduce the secretion level of IL-6 in RAW264.7 cells induced by LPS, RPEO has the strongest inhibitory effect (Fig. 2h). The level of SOD in the cells of the four essential oils increased significantly, and RAEO (50 μg/mL) and RTEO (50 μg/mL) performed better (Fig. 2i).

*3.6. Toxicity*

In the acute toxicity in mice, after oral administration of four *Rhododendron* species essential oils (2000-5000 mg/kg), no mice died or exhibited any acute behavior. The LD_50_ was calculated to be more than 5000 mg/kg. In addition, From Fig. 2j, we can see that for essential oils presented the weak cytotoxicity against RAW 264.7 cells. At the concentration of 250 μg/mL, the cell viability was more than 50.00 %. This result suggested that the four essential oils were safe at the given doses or concentrations (Fig. 2h).

*3.7. The toxicity and anti-inflammatory activity of main compounds*

To find the active compounds of four essential oils, the toxicity and anti-inflammatory activity of seven compounds were studied. In these, phenylethyl alcohol (PA), and 4-hydroxy-3-methylacetophenone (HA) from RPEO, α-pinene (α-PI) and β-pinene from (β-PI) RAEO, β-farnesene (FA) and germacrone (GE) from RTEO, and benzylacetone (BE) from RCEO were chose. As shown in Fig. 3a, as well as the essential oils, seven compounds also have the weak cytotoxicity, and the cell viability was more than 50 % at the concentration of 250 μg/mL. Subsequently, the NO content in RAW 264.7 cells was determined to indirectly explain the anti-inflammatory of compounds. As shown in Fig.3b, except PA, other compounds presented the significant inhibitory effect on NO production compared with the model group. This result indicated that except PA, those compositions in essential oils played the synergistic effect to contribute their anti-inflammatory activity. However, the active compound of RPEO should be studied further.

**4.Discussion**

Currently, high-quality CO_2_-SFE is widely used to extract essential oils from medicinal plants. Compared with traditional hydrodistillation, this technique could improve the yield of essential oils and reduce the cost, avoid the danger of flammability and explosion during traditional solvent extraction, and maintain some thermolability components (17). Therefore, SFE using CO_2_ is an appropriate choice to manufacture essential oil products considering the lipophilic characteristics of essential oils (18). The essential oils components from the same plant depend on multiple factors, such as climate, soil condition, geographical location, collection time and extraction methods, as well as analytical procedures, which have been demonstrated by compelling evidence (21). Headspace solid-phase microextraction (HS-SPME) technology with simple sample pretreatment process, high sensitivity and relatively noninvasive nature, HS-SPME has an enrichment effect on substances (19, 20), and many more compounds have been identified, which may also be one of the reasons why it is different from previous studies. Hence, more compounds were identified from these plants in our study than in previous studies (9).

A previous study reported that the essential oils of *R. thymifolium*, *R. capitatum* and *R. anthopogonoides* isolated using the steam distillation method have strong antibacterial activity against *S. aureus* but have no activity against *E. coli*, *P. aeruginosa* or *C. albicans* (9). *P. aeruginosa* has high internal resistance to almost all known antibiotics and antimicrobials, even synthetic drugs, owing to a very restrictive outer membrane barrier, which has been a serious problem worldwide (22). Interestingly, different from previous studies, four essential oils showed inhibitory effect on *P. aeruginosa*, which indicated that RAEO has the potential to become an alternative antibiotic agent for preventing *P. aeruginosa* infection.

Animal acariasis can reduce the production and quality of animal products and is often fatal. Our previous study showed that *R. nivale* oil had *in vitro* acaricidal activity against adult *P. cuniculi* in a concentration- and time-dependent manner. As a main and active compound, γ-cadinene presented marked acaricidal activity against *Psoroptes cuniculi* (10). Consistent with the previous studies of other Rhododendron, the essential oils of this four have certain acaricidal activity though not very prominent.

More than 50% of medicinal rhododendron species used as folk medicines have been used to treat inflammation-related ailments such as arthritis, rheumatoid diseases, and bronchitis (5). In this study, we studied and compared the anti-inflammatory activities of the four essential oils. First, carrageenan-induced mouse paw edema was used to evaluate the inhibitory activity, which is a biphasic event, and early phase hyperemia was related to the release of histamine, serotonin and similar substances. The later phase was associated with the activation of kinin-like substances (23). Inflammation is an important part of immunopathogenesis. RAW264.7 cells, activated by LPS, release a variety of inflammatory factors, including NO and IL-6. NO is essential for host innate immune responses to pathogens, and excessive NO brings about the development of inflammatory diseases. Therefore, inhibiting the production of NO is the primary target of anti-inflammatory drug development (24). IL-6 is a pleiotropic cytokine, the role of which in the acute inflammatory response is mainly manifested in its pro-inflammatory effect on a variety of cells (25). In addition, SOD plays a protective role in various inflammatory diseases, and treatment with SOD mimetics reduces proinflammatory cytokine production (26). The results of anti-inflammatory indicated that the four essential oils presented the anti-inflammatory activity via decreasing the content of NO and IL-6, and activating SOD activity.

Meanwhile, the toxicity and anti-inflammatory activity of main compounds indicated that except PA, those compositions in essential oils played the synergistic effect to contribute their anti-inflammatory activity. However, the active compound of RPEO should be studied further.

**5. Conclusion**

In summary, the supercritical extraction combined HS-SPME-GC-MS analysis could be used to find more compositions from essential oils. The essential oils of four *Rhododendrons* species have the anti-inflammatory and acaricidal activities, and also presented the antibacterial activity against *S. aureus* and *P. aeruginosa.* The mechanism of action of these essential oils against inflammatory should be studied further. These results indicated that four essential oils have certain medicinal value and can be developed as raw materials for the pharmaceutical and perfume industries, and play a guiding role in the development and utilization of the plant.

**ACKNOWLEDGEMENTS**

This work was financed by the National Natural Science Foundation of China (31772790).

**CRediT authorship contribution statement**

Xiaofei Shang: Conceptualization, Writing-original draft. Lixia Dai, Jian He, Xiaorong Yang: Investigation. Jian He: Soft. Jian He: Validation, Data analysis. Bing Li, Xiaorong Yang: Methodology. Jiyu Zhang, Yanming Wei: Supervision, Project administration.

**Declaration of Competing Interest**

The authors have no competition and no conflicts of interest.

**References**

1. Costa, W.K., de Oliveira, J.R.S., de Oliveira, A.M., da Silva Santos, I.B., da Cunha, R.X., de Freitas, A.F.S., da Silva, J.W.L.M., Silva, V.B.G., de Aguiar, J.C.R. de O. F., da Silva, A.G., Navarro, D.M. do A.F., de Menezes Lima, V.L., da Silva, M.V. Essential oil from *Eugenia stipitata* McVaugh leaves has antinociceptive, anti-inflammatory and antipyretic activities without showing toxicity in mice, *Ind Crop Prod*. (2020) 144, 112059. doi: 10.1016/j.indcrop.2019.112059

2. Alves-Silva J.M., Guerra I., Gonçalves M.J., Cavaleiro C., Cruz M.T., Figueirinha A, Salgueiro L. Chemical composition of *Crithmum maritimum* L. essential oil and hydrodistillation residual water by GC-MS and HPLC-DAD-MS/MS, and their biological activities. *Ind Crops Prod*. (2020) 149, 112329. doi: 10.1016/j.indcrop.2020.112329

3. Chen, Y., Luo, J., Zhang, N., Yu, W., Jiang, J., Dai, G. Insecticidal activities of *Salvia hispanica* L. essential oil and combinations of their main compounds against the beet armyworm *Spodoptera exigua.* *Ind Crops Prod*. (2021) 162, 113271. doi: 10.1016/j.indcrop.2021.113271

4. Al-Ansari, M.M., Andeejani, A.M.I., Alnahmi, E., AlMalki, R.H., Masood, A., Vijayaraghavan, P., Rahman, A.A., Choi, K.C. Insecticidal, antimicrobial and antioxidant activities of essential oil from *Lavandula latifolia* L. and its deterrent effects on *Euphoria leucographa*. *Ind Crops Prod*. (2021) 170, 113740. doi: 10.1016/j.indcrop.2021.113740

5. Popescu, R., Kopp, B. The genus *Rhododendron*: An ethnopharmacological and toxicological review. *J. Ethnopharmacol.*, (2013) 147(1), 42-62. doi: 10.1016/j.jep.2013.02.022

6. Dai, L., He, J., Miao, X., Guo, X., Shang, X., Wang, W., Li, B., Wang, Y., Pan, H., Zhang, J. Multiple biological activities of *Rhododendron przewalskii* Maxim. extracts and UPLC-ESI-Q-TOF/MS characterization of their phytochemical composition. *Front Pharmacol*. (2021) 12, 599778. doi: 10.3389/fphar.2021.599778

7. Jesionek, A., Kokotkiewicz, A., Mikosik-Roczynsk, A., Ciesielska-Figlon, K., Luczkiewicz, P., Bucinski, A., Daca, A., Witkowski, J.M., Bryl, E, Zabiegala, B, Luczkiewicz, M. Chemical variability of *Rhododendron tomentosum* (*Ledum palustre*) essential oils and their pro-apoptotic effect on lymphocytes and rheumatoid arthritis synoviocytes. *Fitoterapia*. (2019) 139, 104402. doi: 10.1016/j.fitote.2019.104402

8. Qiang, Y., Zhou, B., Gao, K., Chemical constituents of plants from the genus *Rhododendron*. *Chem. Biodiversit*. (2011) 8, 792-815. doi: 10.1002/cbdv.201000046

9. Liang, J., You, C., Guo, S., Zhang, W., Li, Y., Geng, Z., Wang, C., Du, S., Deng, Z., Zhang, J. Chemical constituents of the essential oil extracted from *Rhododendron thymifolium* and their insecticidal activities against *Liposcelis bostrychophila* or *Tribolium castaneum*. *Ind Crops Prod*. (2016) 79, 267-273. doi: 10.1016/j.indcrop.2015.11.002

10. Guo, X., Shang, X., Li, B., Zhou, X.Z., Wen, H., Zhang, J. Acaricidal activities of the essential oil from *Rhododendron nivale* Hook. f. and its main compund, δ-cadinene against *Psoroptes cuniculi*. *Vet Parasitol*. (2017) 236, 51-54. doi: 10.1016/j.vetpar.2017.01.028

11. Delectis Florae Reipublicae Popularis Sinicae Agendae Academiae Sinicae Edita. Flora Reipublicae Popularis Sinicae. *Beijing: Science Press*, (1999) 57(1):180.

12. Liang, J., Yang, Q., Ma, X.M., Du, X.Y., Zhang, J. The content and antibacterial activity of volatile oil from three species of *Rhododendron* in Gansu Province. *Chin. Wild Plant Res*. (2014) 33, 9-10.

13. Shao, Q., Deng, Y., Liu, H., Zhang, A., Huang, Y., Xu, G., Li M. Essential oils extraction from *Anoectochilus roxburghii* using supercritical carbon dioxide and their antioxidant activity. *Ind Crops Prod*. (2014) 60, 104-112. doi: 10.1016/j.indcrop.2014.06.009

14. Dai, L., Li, J.C., Miao, X.L., Guo, X., Shang, X.F., Wang, W.W., Li, [B.](https://www.sciencedirect.com/science/article/abs/pii/S0926669021000522#!), Wang, Y., Pan, H., Zhang, J.Y. Ultrasound-assisted extraction of five anthraquinones from *Rheum palmatum* water extract residues and the antimicrobial activities. *Ind. Crop. Prod*. (2021) 162, 223288. doi: 10.1016/j.indcrop.2021.113288

15. Shang, X., Miao, X., Dai, L., Wang, Y, Li, B., Pan, H., Zhang, J.Y. Acaricidal activity of strophanthidin derivatives against *Psoroptes cuniculi* and their inhibitory effect on Na+-K+-ATPase. *Vet Parasitol*. (2021) 296, 109498. doi: 10.1016/j.vetpar.2021.109498

16. Shang, X., Wang, J., Li, M., Miao, X., Pan, H., Yang, Y., Wang, Y. Antinociceptive and anti-inflammatory activities of *Phlomis umbrosa* Turcz extract. *Fitoterapia*. (2011) 82(4), 716-21. doi: 10.1016/j.fitote.2011.03.001

17. Fornari, T., Vicente, G., Vázquez, E., García-Risco, M.R. Reglero, G. Isolation of essential oil from different plants and herbs by supercritical fluid extraction. *J. Chromatogr. A*. (2012) 1250, 34-48. doi: 10.1016/j.chroma.2012.04.051

18. Anitescu, G., Doneanu, C., Radulescu, V. Isolation of coriander oil: comparison between steam distillation and supercritical CO_2_ extraction. *Flav. Frag. J*. (1997) 12(3), 173-176. doi: 10.1002/(SICI)1099-1026(199705)12:3&lt;173::AID-FFJ630&gt;3.0.CO

19. Baky, M. H., Farag, M. A., Rasheed, D.M. Metabolome-based analysis of herbal cough preparations via headspace solid-phase microextraction GC/MS and multivariate data analyses: a prospect for its essential oil equivalency. *ACS omega*. (2020) 5(48), 31370-31380. doi: 10.1021/acsomega.0c04923

20. Zhang, Q., Zhou, L., Chen, H., Wang, C.Z., Xia, Z., Yuan, C.S. Solid-phase microextraction technology for in vitro and in vivo metabolite analysis. *TRAC*. (2016) 80, 57-65. doi: 10.1016/j.trac.2016.02.017

21. Aziz, Z.A.A., Ahmad, A., Setapar, S.H.M., Karakucuk, A., Azim, M.M., Lokhat, D., Rafatullah, M., Ganash, M., Kamal, M.A., Ashraf, G.M. Essential oils: extraction techniques, pharmaceutical and therapeutic potential-A review. *Curr Drug Metab*. (2018) 19(13), 1100-1110. doi: 10.2174/1389200219666180723144850

22. Hayakawa, S., Kawamura, M., Sato, T., Hirano, T., Fujimura, S. An α-lipoic acid derivative, and anti-ros agent, prevents the acquisition of multi-drug resistance in clinical isolates of *Pseudomonas aeruginosa*. *J. Infect. Chemother*. (2018) 25(1), 28-33. doi: 10.1016/j.jiac.2018.10.003

23. Li, M., Shang, X., Zhang, R., Jia, Z., Fan, P., Ying, Q., Wei, L. Antinociceptive and anti-inflammatory activities of iridoid glycosides extract of *Lamiophlomis rotata* (Benth.) Kudo. *Fitoterapia*. (2010) 81(3), 167-72. doi: 10.1016/j.fitote.2009.08.018

24. Yoon, S.B., Lee, Y. J., Park, S.K., Kim, H.C., Bae, H., Kim, H.M., Ko, S.G., Choi, H.Y., Oh, M.S., Park, W. Anti-inflammatory effects of *Scutellaria baicalensis* water extract on LPS-activated raw 264.7 macrophages. *J. Ethnopharmacol*. (2009) 125(2), 286-290. doi: 10.1016/j.jep.2009.06.027

25. Tilg, H., Trehu, E., Atkins, M.B., Dinarello, C.A., Mier, J.W. Interleukin-6 (il-6) as an anti-inflammatory cytokine: induction of circulating il-1 receptor antagonist and soluble tumor necrosis factor receptor p55. *Blood*. (1994) 83(1), 113-118. doi: 10.1182/blood.V83.1.113.bloodjournal831113

26. Yasui, K., Baba, A. Therapeutic potential of superoxide dismutase (sod) for resolution of inflammation. *Inflamm. Res*. (2006) 55(9), 359. doi: 10.1007/s00011-006-5195-y

**Figures and Tables Legends**

Figure 1. The yields of four essential oils (a) and venn picture of common compositions of four essential oils (b).

Figure 2. The paw edema level (a) and its inhibition level (b) of carrageenan-induced paw edema in mice test, and the ear edema level (c) and its inhibition level (d) of xylene-induced ear edema test, the inhibitory effect of these compounds on NO production (e), IL-6 production (f) and SOD activity (g) in RAW264.7 cells induced by LPS, the cell viability of four *Rhododendrons* species essential oils against RAW264.7 cells.

Figure 3. The cell viability of seven components from four *Rhododendrons* species (a) and the inhibitory effect of these compounds on NO production in RAW264.7 cells induced by LPS (b).

Figure S1. Total ion chromatogram of essential oils from four species.

Table 1. The top 30 compounds of the essential oils from four *Rhododendron* species

Table 2. The antibacterial activity of the essential oils from four *Rhododendron* species

Table 3. The acaricidal activity of the essential oils from four *Rhododendron* species

Table S1. The identified compounds of the essential oils from four *Rhododendron* species

Table S2. The common compounds of the essential oils from four *Rhododendron* species


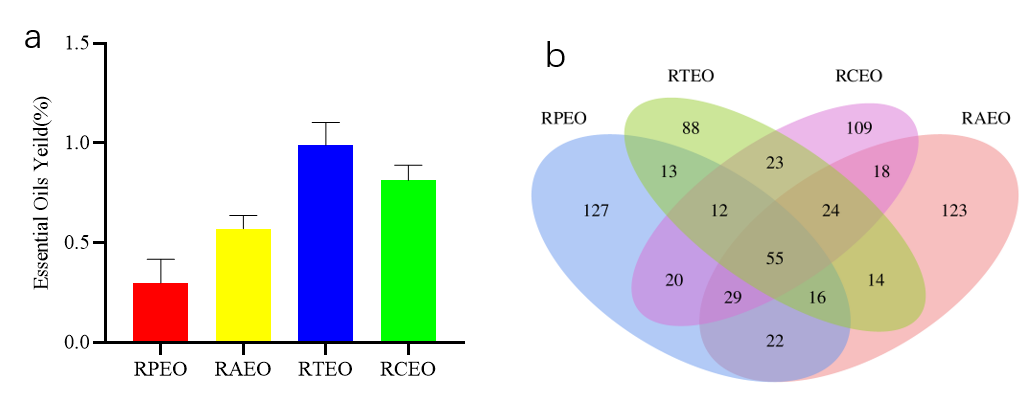
Figure 1. The yields of four essential oils (a) and venn picture of common compositions of four essential oils (b).


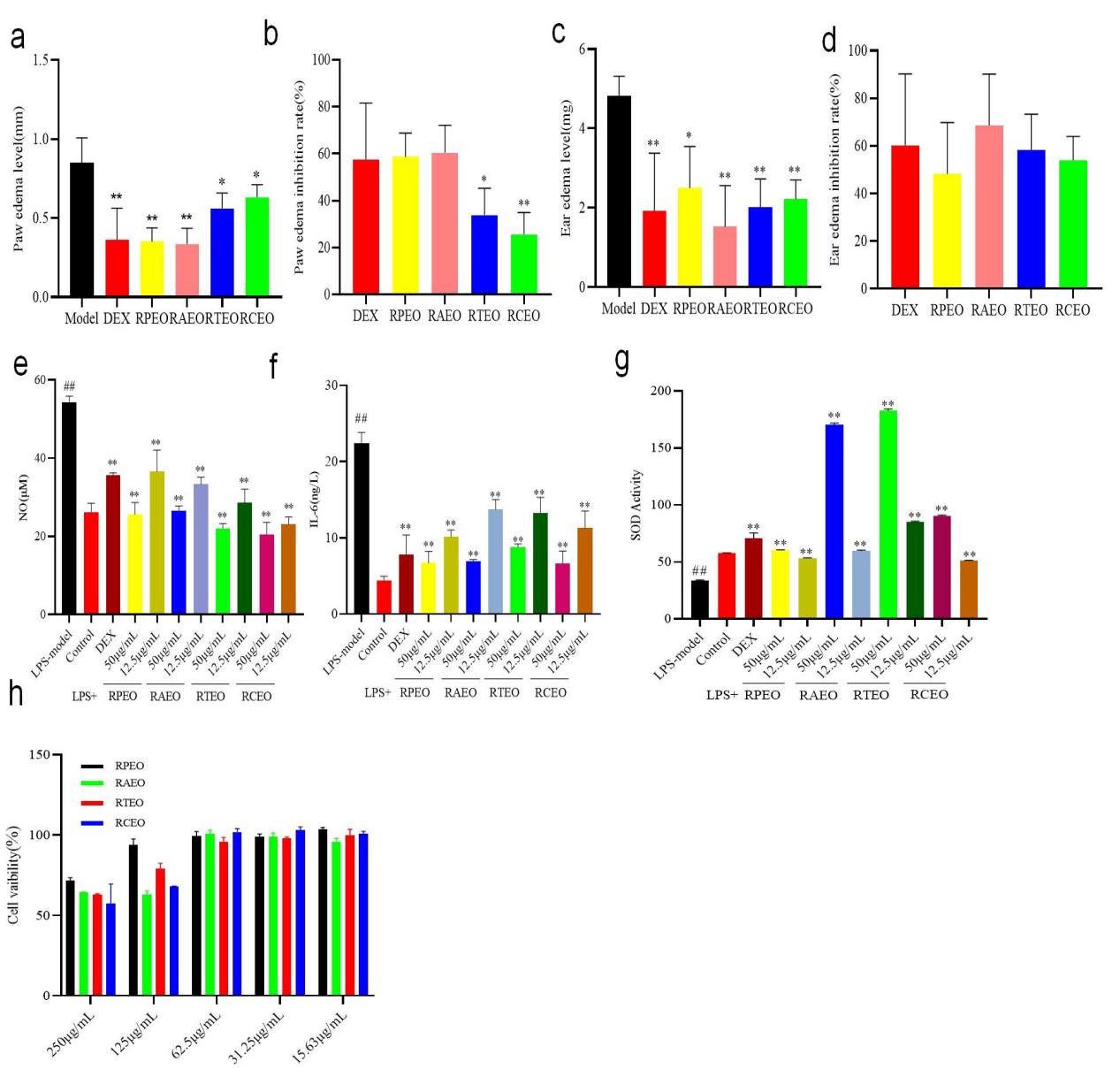


Figure 2. The paw edema level (a) and its inhibition level (b) of carrageenan-induced paw edema in mice test, and the ear edema level (c) and its inhibition level (d) of xylene-induced ear edema test, the inhibitory effect of these compounds on NO production (e), IL-6 production (f) and SOD activity (g) in RAW264.7 cells induced by LPS, the cell viability of four *Rhododendrons* species essential oils against RAW264.7 cells. (^##^represent the significant difference between control group and model group, *p<0.01;* * *p<0.05* represent the significant difference between model group and drug-treated group; ** for *p<0.01*)

a

b

Figure 3. The cell viability of seven components from four *Rhododendrons* species (a) and the inhibitory effect of these compounds on NO production in RAW264.7 cells induced by LPS (b). (phenylethyl alcohol (PA), and 4-hydroxy-3-methylacetophenone (HA) from RPEO(essential of *Rhododendrron* *przewalskii* Maxim.), α-pinene (α-PI) and β-pinene (β-PI) from RAEO(essential oil of *Rhododendron anthopogonoides* Maxim.), β-farnesene (FA) and germacrone (GE) from RTEO(essential oil of *Rhododendron thymifolium* Maxim.), and benzylacetone (BE) from RCEO (essential oil of *Rhododendron* *capitatum* Maxim.) (^##^represent the significant difference between control group and model group, *p<0.01;* * *p<0.05* represent the significant difference between model group and drug-treated group; ** for *p<0.01*)
