# Supplementary material for: Chemical constituents, antibacterial, acaricidal and anti-inflammatory activities of the essential oils from four Rhododendron species
Source: Front Vet Sci. 2022 Aug 10;9:882060. doi: 10.3389/fvets.2022.882060 (PMC9399923; doi:10.3389/fvets.2022.882060)
Supplement: Supplementary Table S1 — The identified compounds of the essential oils from four Rhododendron species. [file Data_Sheet_1.zip › 22-7-24/Total ion chromatogram/Figure s1.docx]

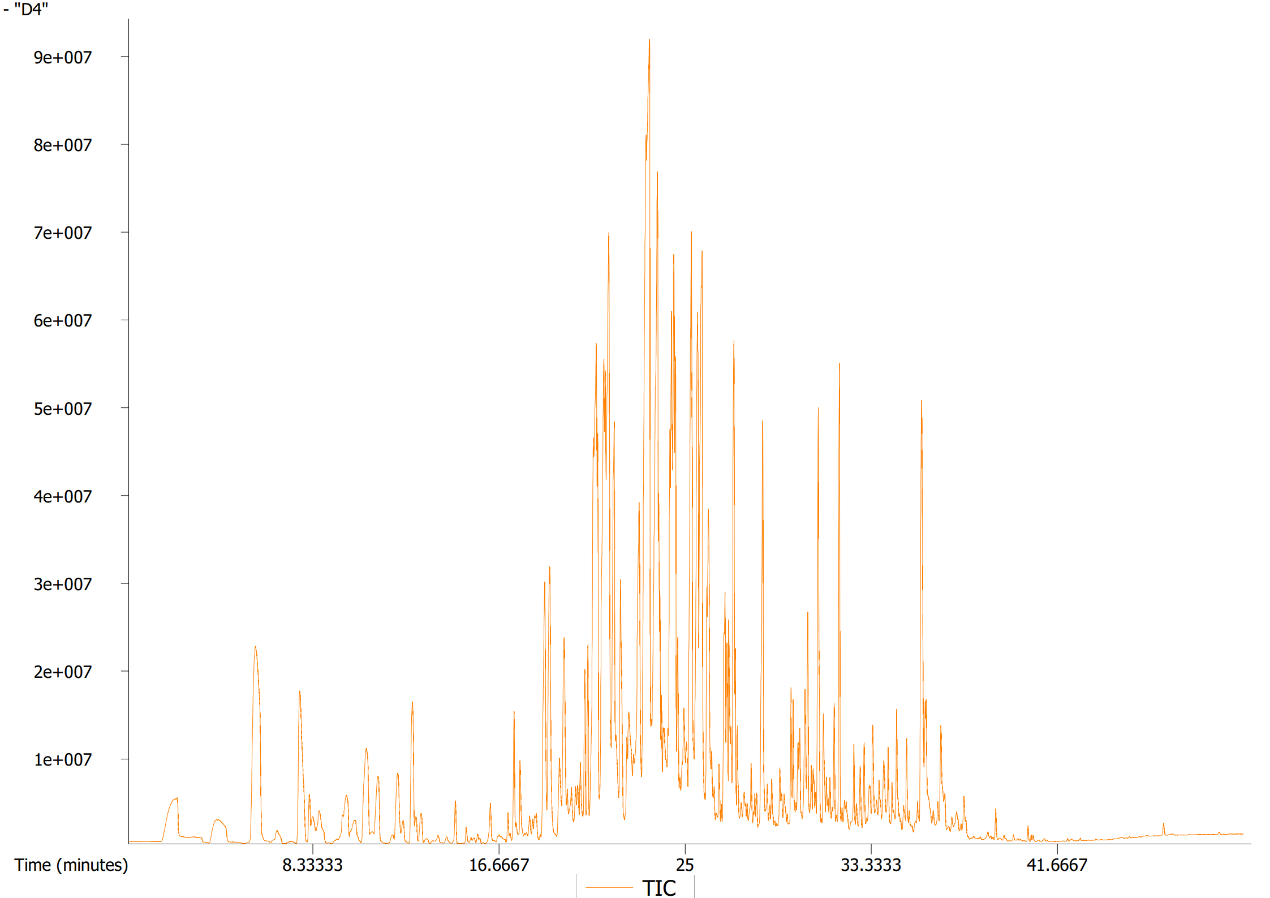


Total ion chromatogram of essential oils from *Rhododendron anthopogonoides* Maxim.


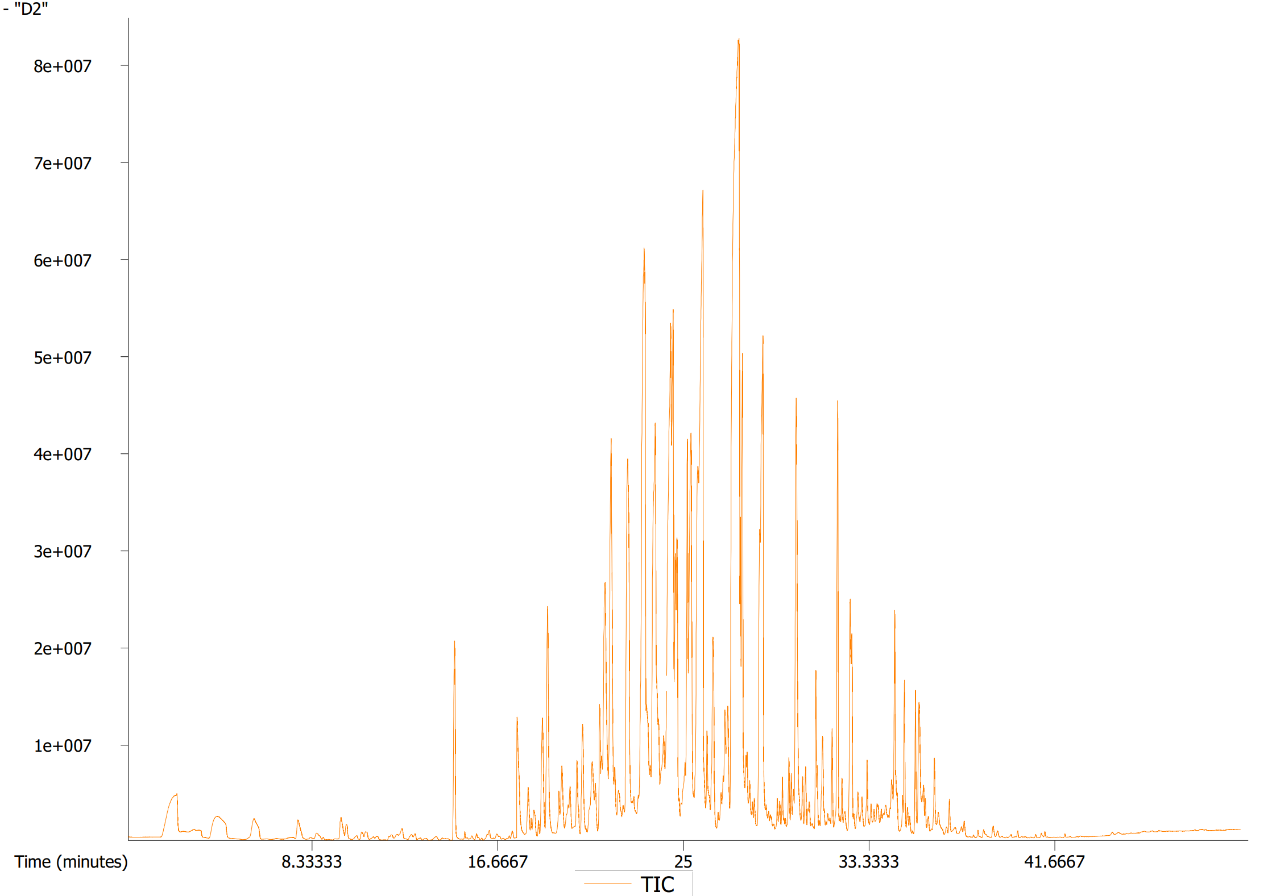


Total ion chromatogram of essential oils from *Rhododendron capitatum* Maxim.


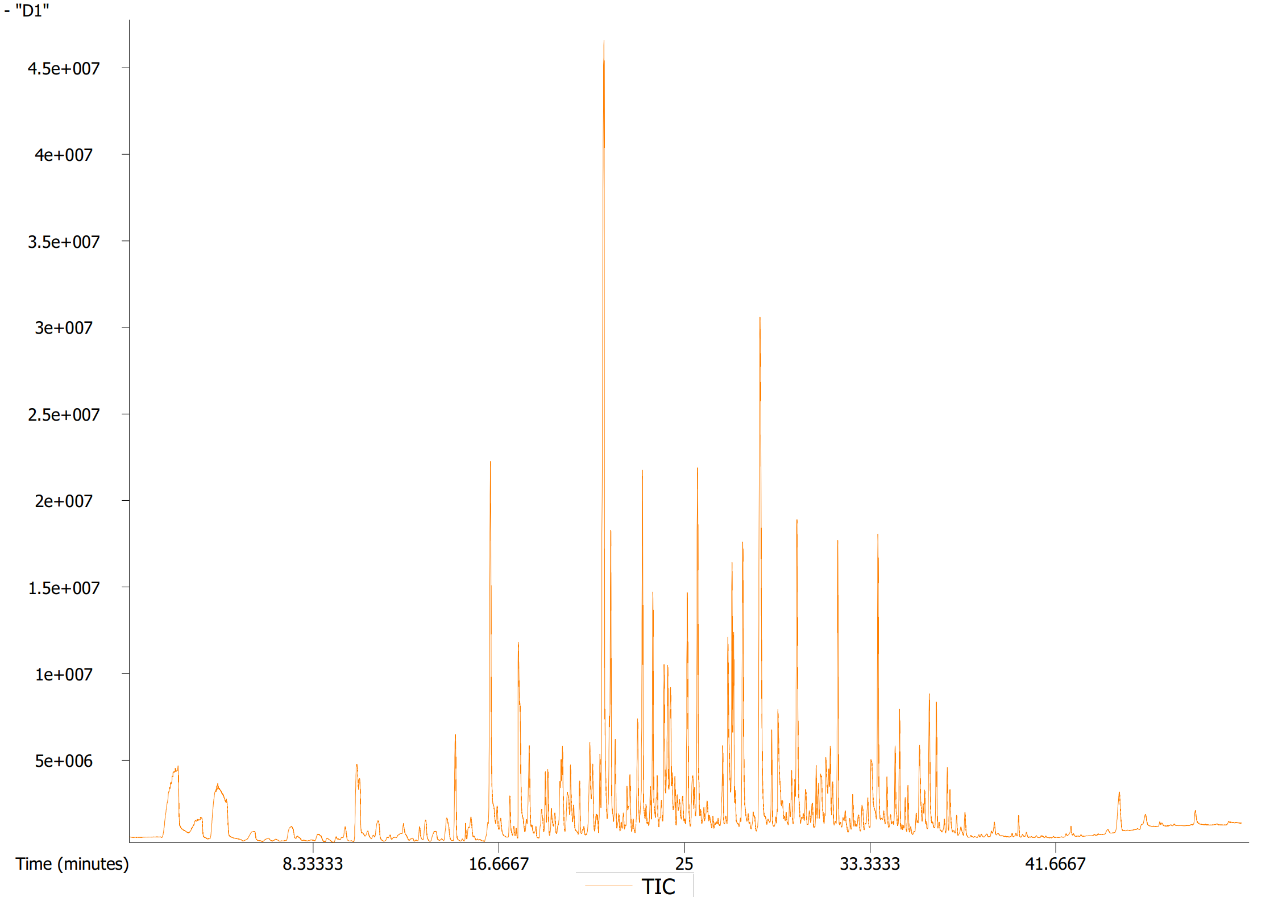


Total ion chromatogram of essential oils from *Rhododendron przewalskii* Maxim.


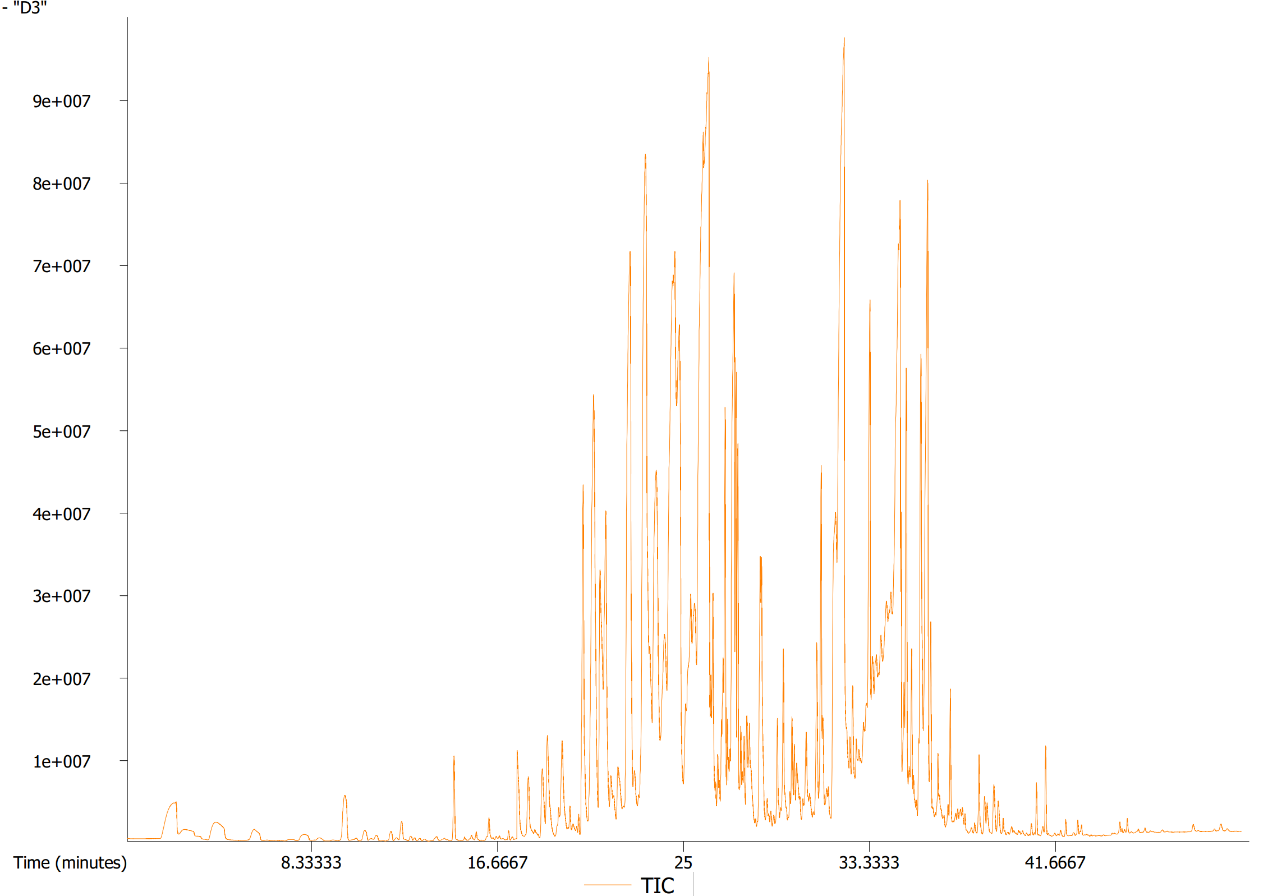


Total ion chromatogram of essential oils from *Rhododendron thymifolium* Maxim.
