# Supplementary material for: Chemical constituents, antibacterial, acaricidal and anti-inflammatory activities of the essential oils from four Rhododendron species
Source: Front Vet Sci. 2022 Aug 10;9:882060. doi: 10.3389/fvets.2022.882060 (PMC9399923; doi:10.3389/fvets.2022.882060)
Supplement: Supplementary Table S1 — The identified compounds of the essential oils from four Rhododendron species. [file Data_Sheet_1.zip › 22-7-24/╘¡╩╝╩2╛▌/Acaricidal activity/╦─╓╓╗╙╖ó╙═LC50.docx]

样品名称陇蜀杜鹃

回归方程 y(Probit)=-1.1012+1.8868Log(D)

半数致死量 LD50=1712.3

LD50(Feiller校正)95%的可信限=1212.1--4225.8

LD5=230.04

LD95=12745

样品名称烈香杜鹃

回归方程 y(Probit)=-1.8715+2.1467Log(D)

半数致死量 LD50=1588.4

LD50(Feiller校正)95%的可信限=1173.5--2973.4

LD5=272.1

LD95=9272.9

样品名称头花杜鹃

回归方程 y(Probit)=0.5459+1.2053Log(D)

半数致死量 LD50=4960.4

LD50(Feiller校正)95%的可信限=2090.9--1.5504E-22

LD5=214.17

LD95=1.1489E5

样品名称千里香杜鹃

回归方程 y(Probit)=-0.098691+1.5122Log(D)

半数致死量 LD50=2353.3

LD50(Feiller校正)95%的可信限=1454.7--35949

LD5=192.28

LD95=28802
