# Supplementary figures and images for: Chemical constituents, antibacterial, acaricidal and anti-inflammatory activities of the essential oils from four Rhododendron species
Source: Front Vet Sci. 2022 Aug 10;9:882060. doi: 10.3389/fvets.2022.882060 (PMC9399923; doi:10.3389/fvets.2022.882060)

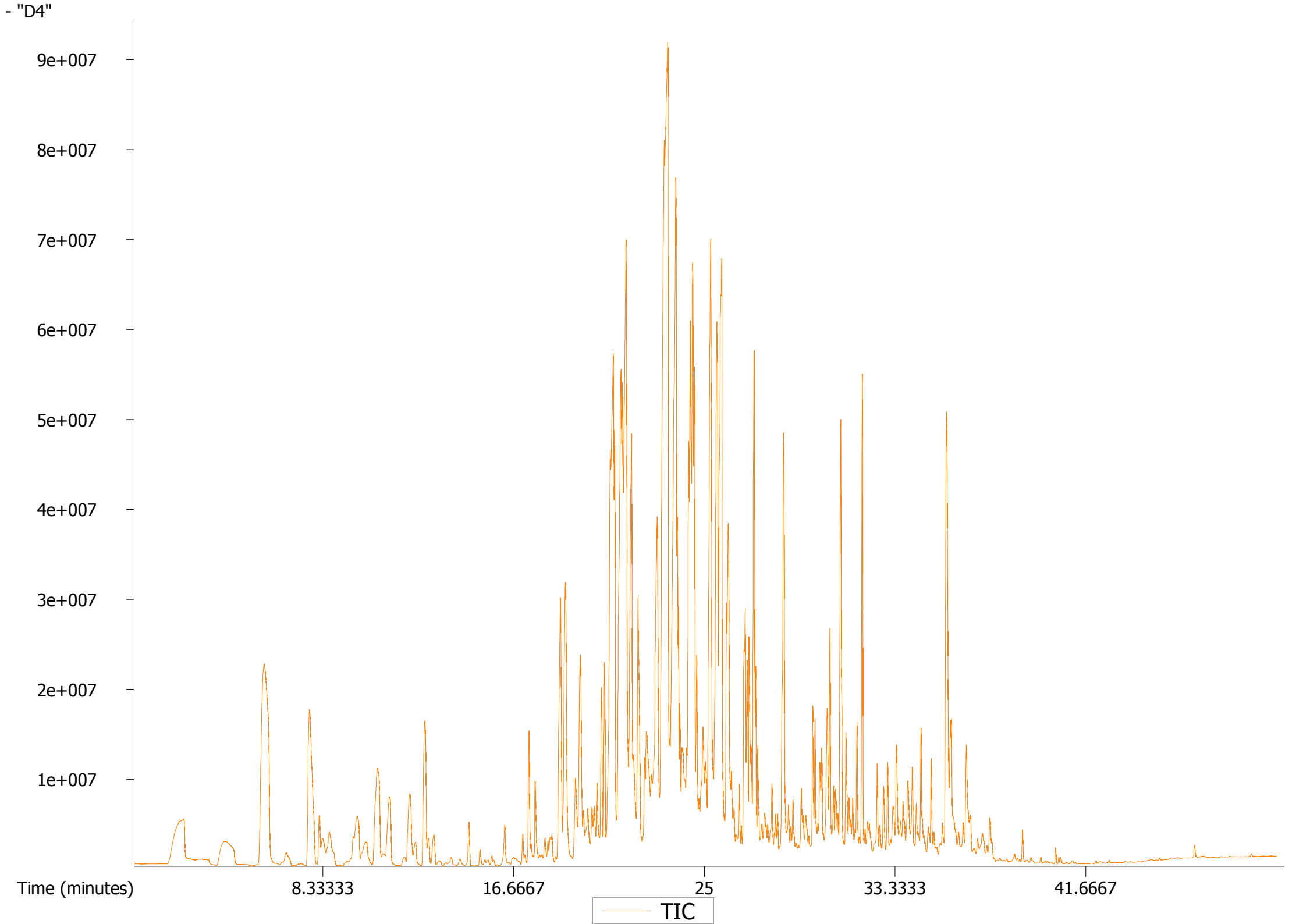

Supplement: Supplementary Table S1 — The identified compounds of the essential oils from four Rhododendron species. [file Data_Sheet_1.zip › 22-7-24/Total ion chromatogram/Rhododendron anthopogonoides Maxim.pdf]

- "D2"

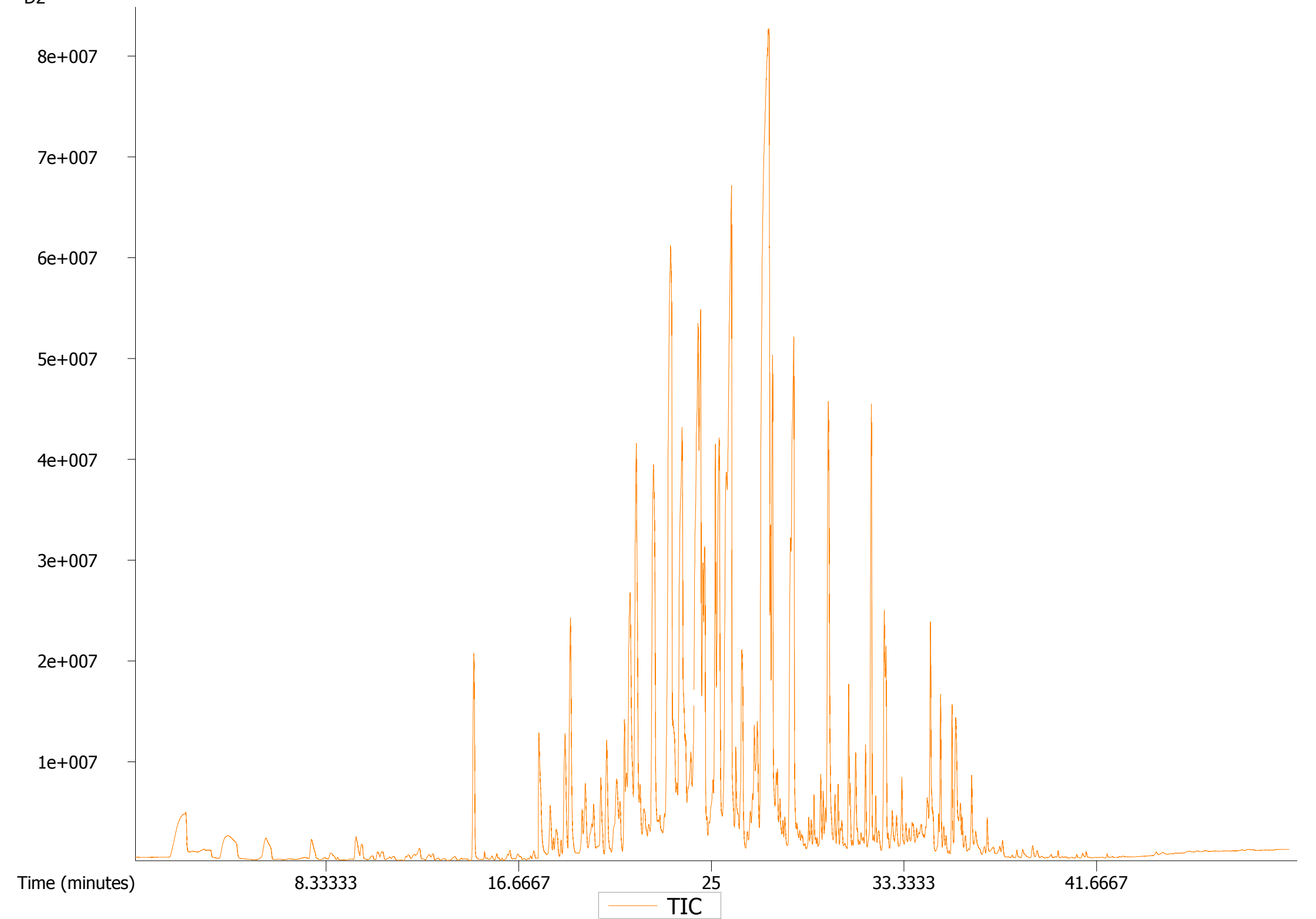

Supplement: Supplementary Table S1 — The identified compounds of the essential oils from four Rhododendron species. [file Data_Sheet_1.zip › 22-7-24/Total ion chromatogram/Rhododendron capitatum Maxim.pdf]

- "D3"

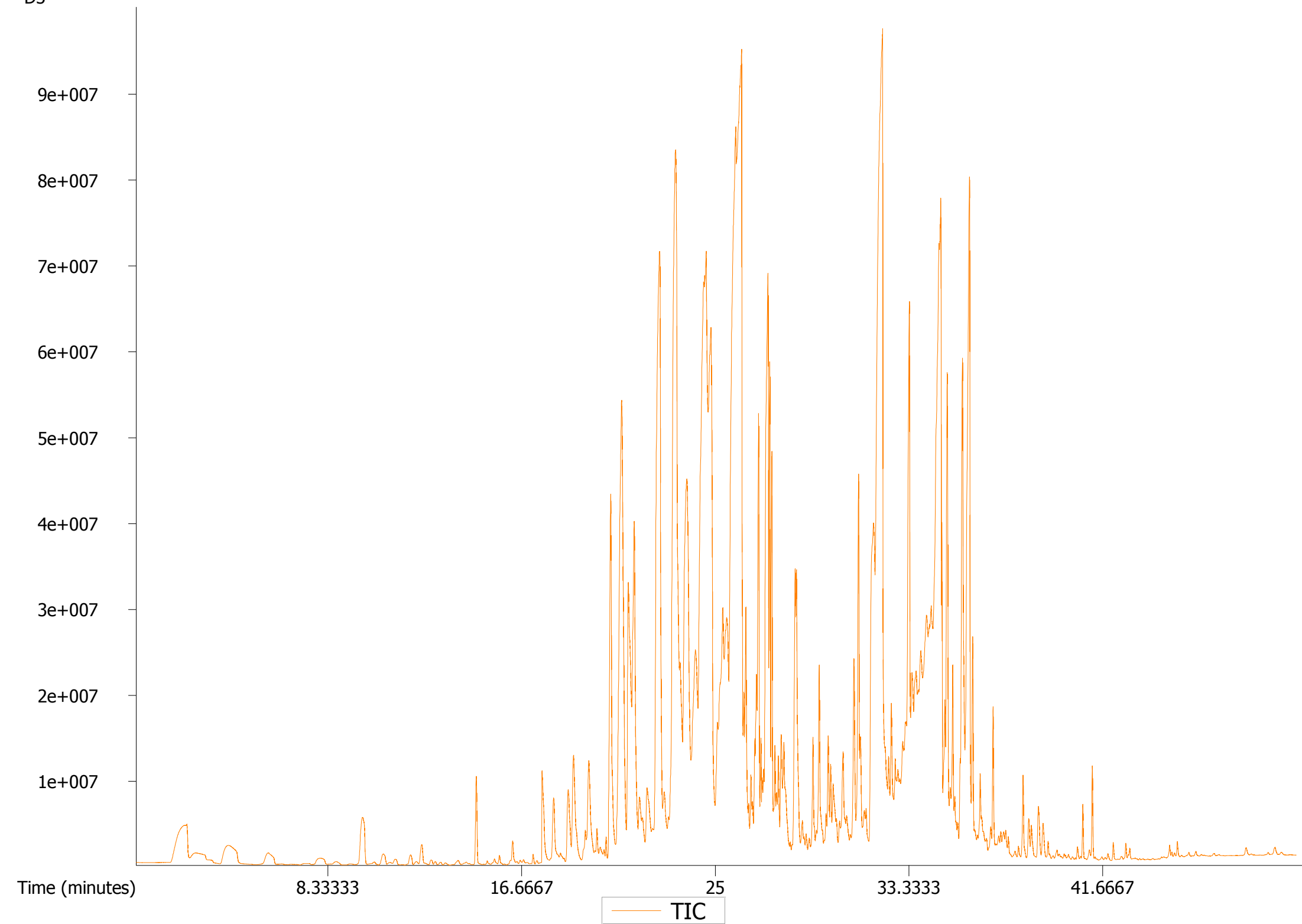

Supplement: Supplementary Table S1 — The identified compounds of the essential oils from four Rhododendron species. [file Data_Sheet_1.zip › 22-7-24/Total ion chromatogram/Rhododendron thymifolium Maxim.pdf]

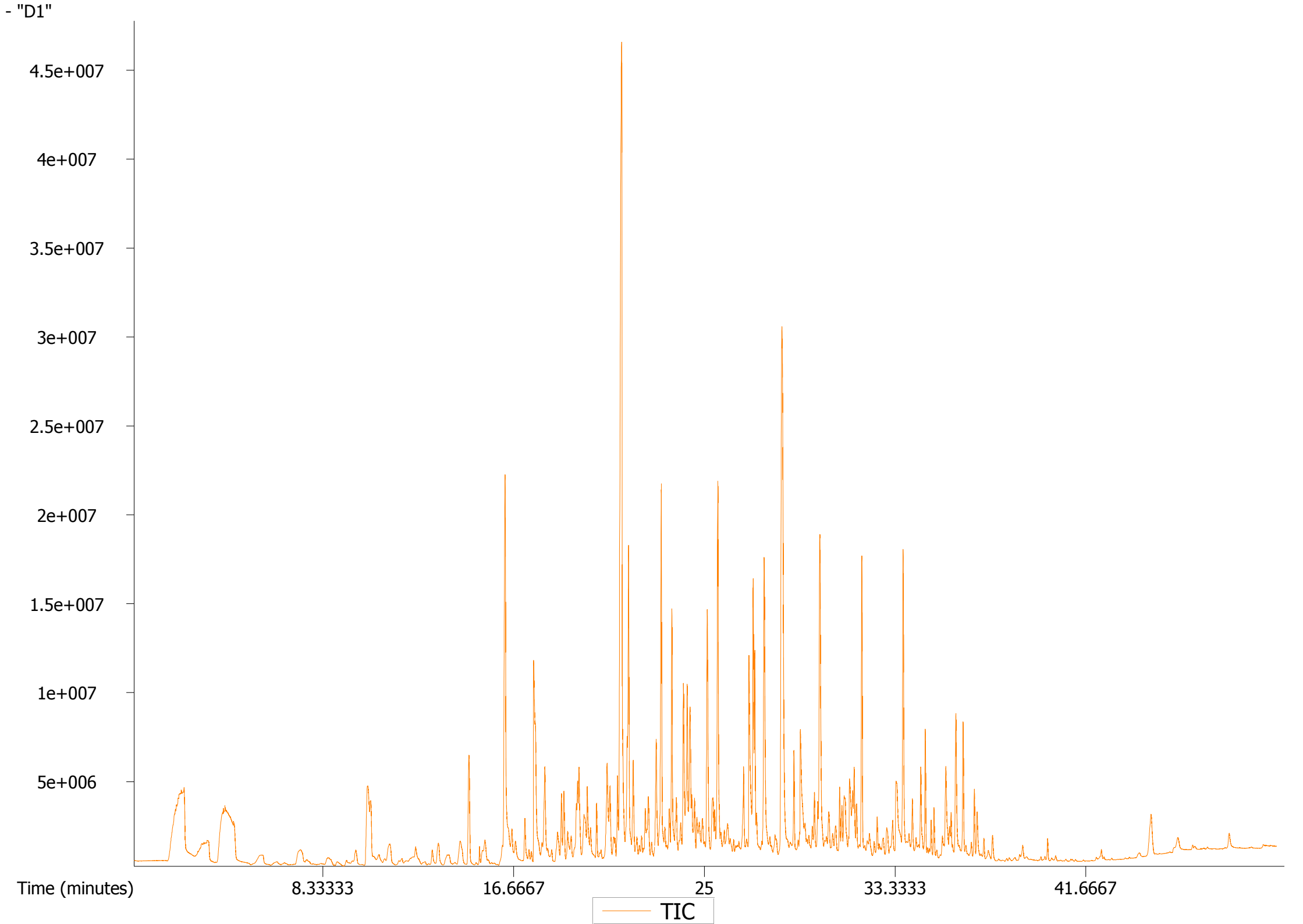

Supplement: Supplementary Table S1 — The identified compounds of the essential oils from four Rhododendron species. [file Data_Sheet_1.zip › 22-7-24/Total ion chromatogram/Rhododendrron przewalskii Maxim.pdf]
