# Supplementary material for: Chemical constituents, antibacterial, acaricidal and anti-inflammatory activities of the essential oils from four Rhododendron species
Source: Front Vet Sci. 2022 Aug 10;9:882060. doi: 10.3389/fvets.2022.882060 (PMC9399923; doi:10.3389/fvets.2022.882060)
Supplement: Supplementary Figure S1 — Total ion chromatogram of essential oils from R. anthopogonoides, R. capitatum, R. przewalskii, and R. thymifolium. [file Table_1.DOCX]

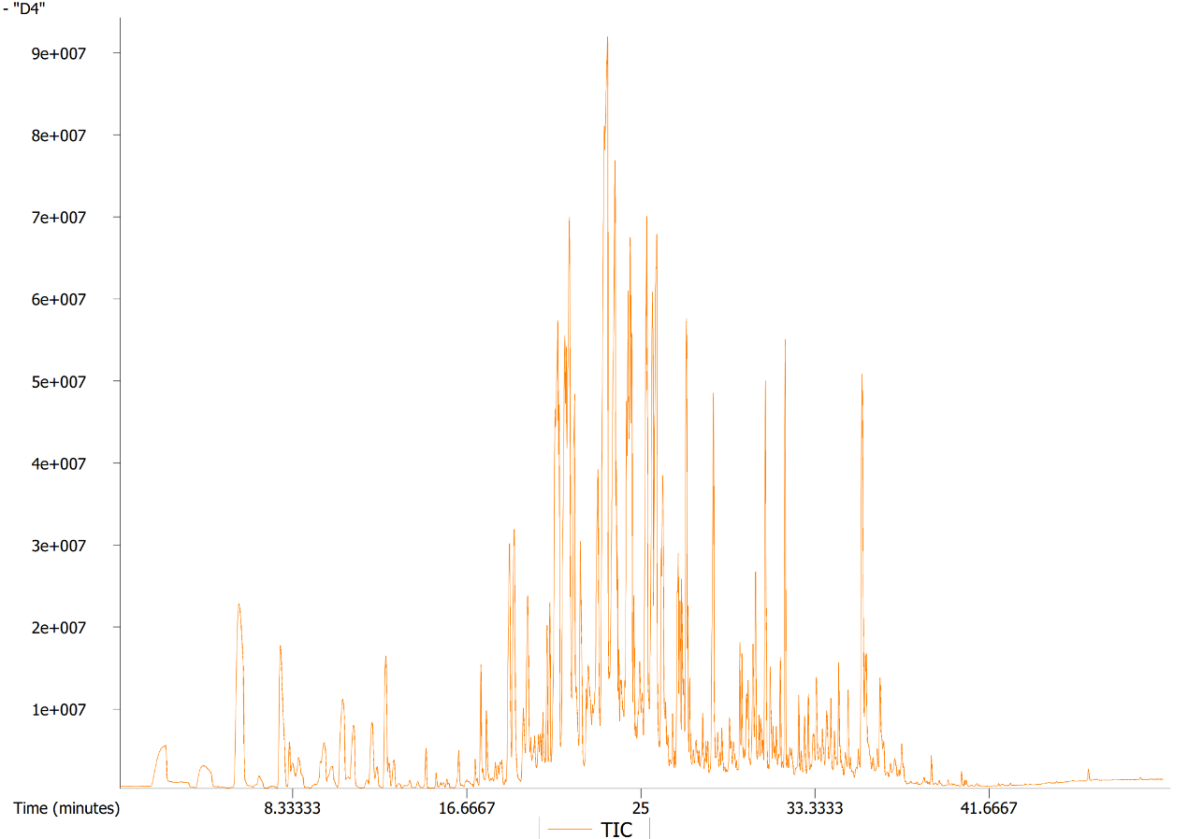
a


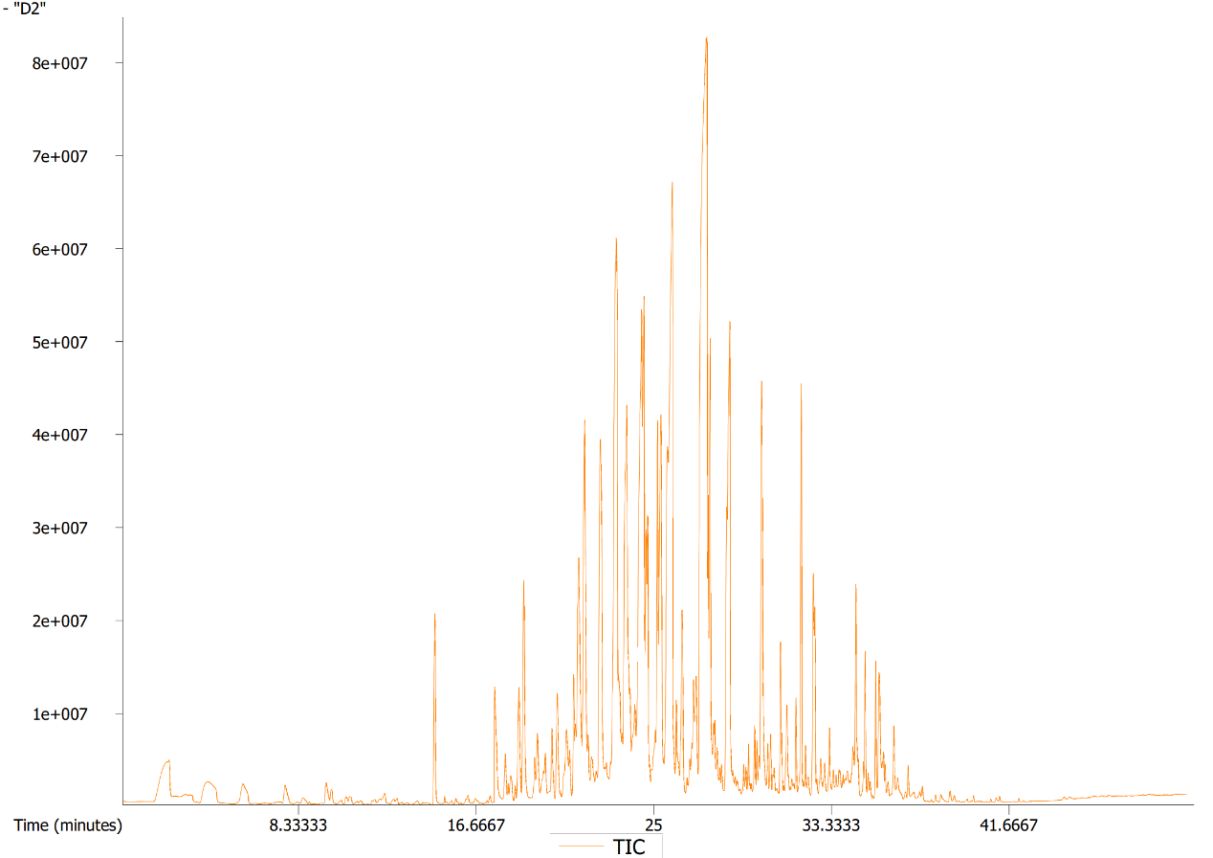
b


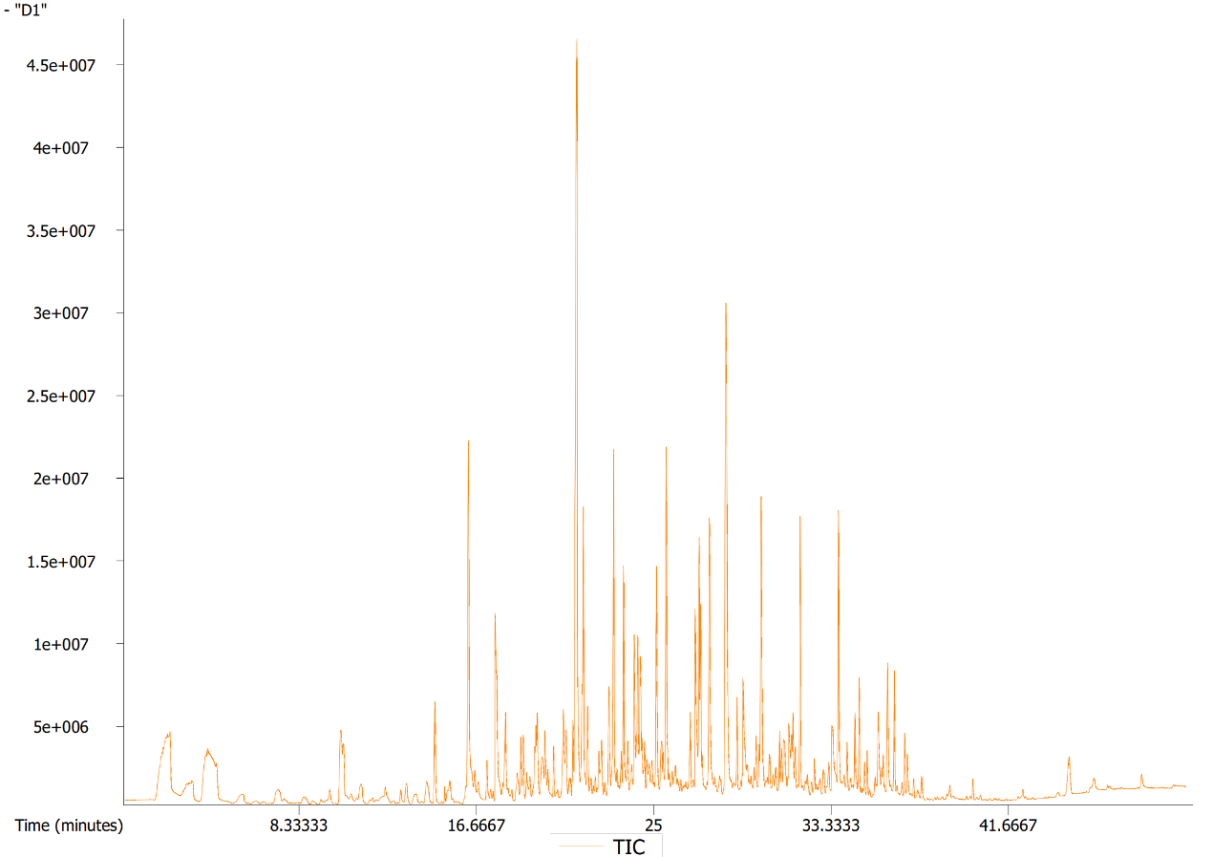
c


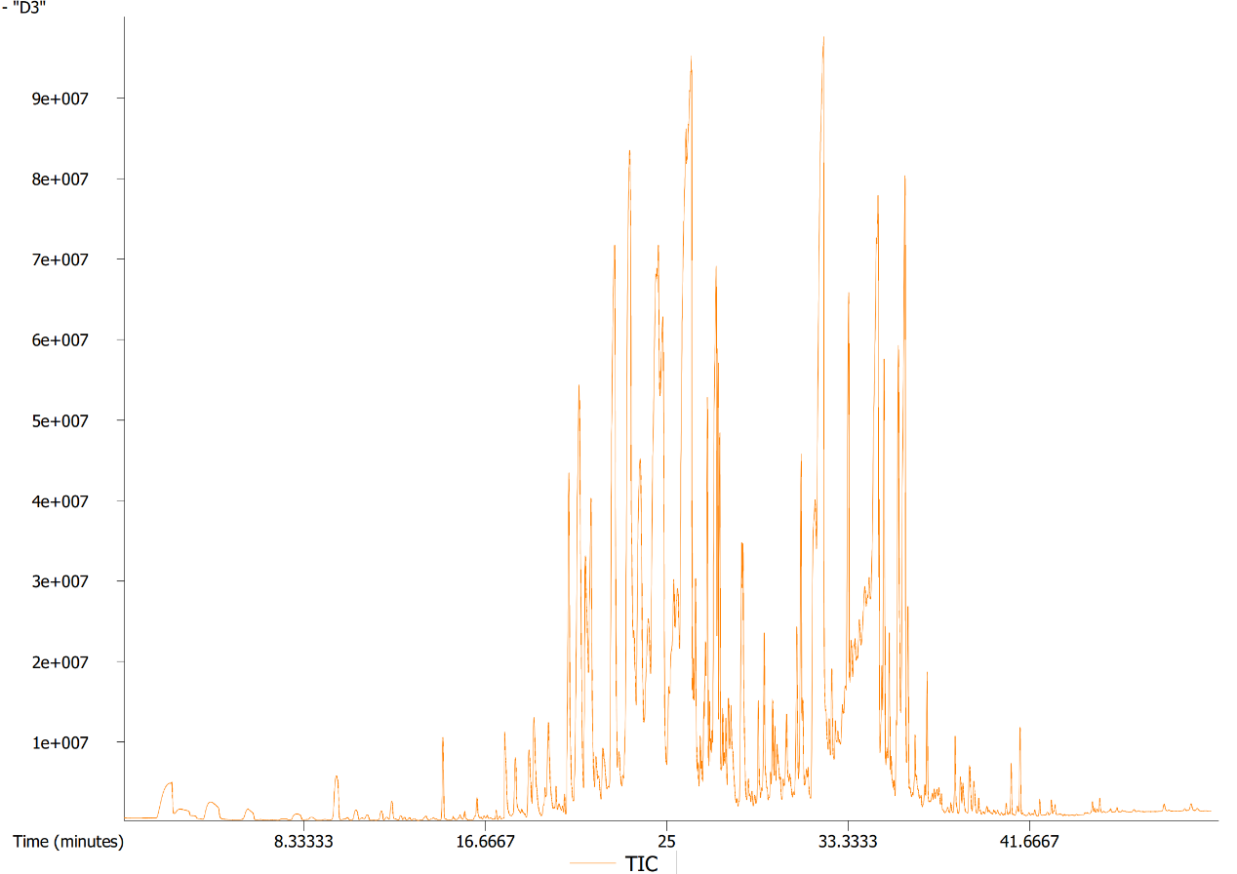
d

Figure S1: Total ion chromatogram of essential oils from *R. anthopogonoides, R. capitatum, R. przewalskii,* and *R. thymifolium*
